# Oxidative stress and counteracting mechanisms in hormone receptor positive, triple-negative and basal-like breast carcinomas

**DOI:** 10.1186/1471-2407-11-262

**Published:** 2011-06-21

**Authors:** Peeter Karihtala, Saila Kauppila, Ylermi Soini, Arja-Jukkola-Vuorinen

**Affiliations:** 1 Department of Oncology and Radiotherapy, Oulu University Hospital and University of Oulu, Oulu, Finland; 2 Department of Pathology, Oulu University Hospital and University of Oulu, Oulu, Finland; 3 Department of Pathology, Oulu University Hospital and Department of Clinical Pathology and Forensic Medicine, Institute of Clinical Medicine, School of Medicine, Cancer Center of Eastern Finland, University of Eastern Finland, Kuopio, Finland

## Abstract

**Background:**

Triple-negative breast cancer (TNBC) and basal-like breast cancer (BLBC) are breast cancer subtypes with an especially poor prognosis. 8-Hydroxydeoxyguanosine (8-OHdG) is a widely used marker of oxidative stress and the redox-state-regulating enzymes peroxiredoxins (PRDXs) are efficient at depressing excessive reactive oxygen species. NF-E2-related factor 2 (Nrf2) and Kelch-like ECH-associated protein 1 (Keap1) are redox-sensitive transcription factors that regulate PRDX expression. This is the first study to assess oxidative stress and or cell redox state-regulating enzymes in TNBC and BLBC.

**Methods:**

We assessed immunohistochemical expression of 8-OHdG, Nrf2, Keap1, PRDX III and PRDX IV in 79 women with invasive ductal breast carcinomas. Of these tumors, 37 represented TNBC (grade II-III tumors with total lack of ER, PR and human epidermal growth factor receptor 2 [HER2] expression). Control cases (n = 42) were ER-positive, PR-positive and HER2-negative. Of the 37 TNBCs, 31 had BLBC phenotype (TNBC with expression of cytokeratin 5/6 or epidermal growth factor receptor 1).

**Results:**

Patients with TNBC had worse breast cancer-specific survival (BCSS) than the control group (p = 0.015). Expression of 8-OHdG was significantly lower in TNBC than in the non-TNBC group (p < 0.005). 8-OHdG immunostaining was associated with better BCSS (p = 0.01), small tumor size (p < 0.0001) and low grade (p < 0.0005). Keap1 overexpression was observed in the TNBC cohort (p = 0.001) and Keap1-positive patients had worse BCSS than Keap1-negative women (p = 0.014). PRDX IV was overexpressed in the TNBC *vs. *the non-TNBC group (p = 0.022).

**Conclusions:**

Cellular redox state markers may be promising targets when elucidating the pathogenesis of TNBC.

## Background

Breast cancers lacking both estrogen and progesterone receptors (ER and PR), with simultaneous absence of human epidermal growth factor 2 receptor (HER2) are defined as triple-negative breast cancers (TNBCs). Currently there are no targeted therapies available for TNBC and chemotherapy is the only option in both adjuvant and metastatic settings. TNBC has a poor prognosis in terms of disease-free survival and overall survival and it tends to be associated with aggressive and early recurrence [1]. Some TNBCs have a basal-like phenotype, and these basal-like breast cancers (BLBCs) show simultaneous expression of cytokeratin (CK) 5/6 or epidermal growth factor-1 (EGFR-1) [2]. It has been suggested that BLBCs may have a different pathogenesis, originating probably from mammary epithelial luminal progenitor cells [3].

Reactive oxygen species (ROS) are continuously produced in all aerobic organisms as a consequence of aerobic respiration. Although many ROS are vital regulators of signaling pathways, oxidative stress occurs if ROS production exceeds the capacity of the ROS-suppressing machinery, which mainly consists of antioxidant enzymes. Oxidative stress is a potent cause of damage in all cellular macromolecules and it may also lead to carcinogenesis [4]. The hydroxyl radical (•OH) is the most unstable ROS and its interaction with DNA leaves a specific and stable footprint, 8-hydroxydeoxyguanosine (8-OHdG i.e. 8-oxodG), which can be reliably assessed by means of immunohistochemistry, for example.

Peroxiredoxins (PRDXs) I-VI are one of the most important antioxidant enzymes and they also modulate intracellular signaling pathways related to apoptosis and cell proliferation [5]. The main antioxidant function of PRDXs is to reduce peroxides, including H_2_O_2_, to corresponding alcohols and water. If H_2_O_2 _is not reduced and it interacts with transition metals (usually ions of Fe or Cu), •OH and consequently 8-OHdG can be formed. PRDXs are strongly induced in oxidative conditions. This induction is largely mediated by redox-sensitive NF-E2-related factor 2 (Nrf2), which under oxidative stress translocates to the nucleus and attaches to antioxidant response elements (AREs) of antioxidant genes, thus stimulating synthesis of respective proteins [6-8]. Nrf2 is negatively regulated by another redox-sensitive protein, Kelch-like ECH-associated protein 1 (Keap1).

Estrogens are important ROS inducers in ER-positive breast cancer cells [9], although data from clinical series is lacking. Our previous results have suggested that two of the most important regulators of the cellular redox state, PRDX III and PRDX IV, may have special roles in steroid receptor-negative breast cancers [10]. This study was designed to find out whether or not oxidative stress and/or cell redox state-regulating enzymes have special roles in TNBC and BLBC. These breast cancer subtypes are especially aggressive and more accurate prognostic and predictive biomarkers are urgently required.

## Methods

The material consisted of 79 women with local or locally advanced breast cancer from a prospective series at Oulu University Hospital, Oulu, Finland, diagnosed during 2000-2008. All tumors showed invasive ductal histology. The specimens had been fixed in neutral formalin, embedded in paraffin blocks and stored at the Department of Pathology at the same institute. The patients were surgically staged according to the current TNM classification system and the histological degree of tumor differentiation was classified according to the WHO Classification of Tumours [11]. The study was approved by the Local Ethics Committee of the Northern Ostrobothnia Hospital District of Finland.

### Immunohistochemistry and scoring

Paraffin-embedded tissues were first sectioned (4 μm thickness) and placed on SuperFrostPlus glass slides, fixed at 37 °C overnight, and processed further within a few days. The sections were deparaffinized in xylene and then rehydrated in a descending ethanol series, incubated in 10 mM citrate buffer (pH 6.0), boiled in a microwave oven for 10 minutes, and cooled thoroughly at room temperature before adding the primary antibody. Negative controls were prepared by using the same procedure except that the primary antibodies were replaced by PBS and serum isotype controls (Zymed Laboratories, Inc.). Previously known positive control samples were also used. Table 1 shows more details of the staining for each antibody used.

**Table 1:** Immunohistochemical methods used in this study.

Antibody (Clone/Product code)	Dilution	Immunostaining method	Source of primary antibody
CK5/6 (D5/16 B4)	1:200	Dako Envision Kit	DakoCytomation, Glostrup, Denmark

EGFR (NCL-L-EGFR-384)	1:200	Dako Envision Kit	Leica Biosystems, Newcastle, United Kingdom

ER (NCL-ER-6F11)	1:50	Dako Envision Kit	Novocastra, Newcastle upon Tyne, United Kingdom

HER2 (NCL-CB11)	1:500	Dako Envision Kit	Novocastra

PR (PgR 636)	1:150	Dako Envision Kit	DakoCytomation

PRDX III (LF-P A 0030)	1:500	Histostain-Plus Bulk Kit	Labfrontier, York, United Kingdom

PRDX IV (LF-P A 0009)	1:500	Histostain-Plus Bulk Kit	Labfrontier

8-OHdG (N45.1)	1:50	Dako Envision Kit	JaICA, Fukuroi, Japan

Nrf2 (SC-722)	1:100	Histostain Plus Broad Spectrum Kit	Santa Cruz Biotechnology, Santa Cruz, USA

Keap1 (SC-15246)	1:100	Biocare Medical HRP Polymer Kit	Santa Cruz

Tumors exhibiting *nuclear *estrogen/progesterone receptor expression in more than 10% of invasive tumor cells were considered as steroid receptor-positive. The TNBC group did not show any ER- or PR-positivity. Membranous HER2 expression was also studied by means of immunohistochemistry (IHC) and if a specimen exhibited a HER2-positive result (1+ to 3+ on a scale of 0 to 3+) in IHC, Her2 gene amplification status was determined by means of chromogenic *in situ *hybridization (CISH). Breast cancers with six or more gene copies of Her2 in cells were considered HER2-positive [12]. Expression of Ki-67 was studied immunohistochemically as described previously, the cut-off for negativity being <5% [10]. Cytokeratin 5/6 was scored positive if any (weak or strong) cytoplasmic and/or membranous invasive carcinoma cell staining was observed and EGFR was scored positive if there were more than 10% of positive cells.

When comparing PRDXs with each other or against tumor parameters or survival, cytoplasmic PRDX immunostaining was divided into two groups, as in our previous breast cancer study [10]: 0 = absent or weak staining and 1 = moderate or strong staining. Since 97.5% of the cases were negative or only weakly positive for nuclear PRDX III, this parameter was divided into either negative or any amount of positive staining for further statistical analyses. Interpretation of 8-OHdG immunostaining was similar to that used for cytoplasmic PRDXs, but only nuclear 8-OHdG staining was evaluated. In the case of Nrf2, nuclear immunopositivity, and in the case of Keap1 both nuclear and cytoplasmic immunopositivity were considered as a positive result.

Immunostaining of 8-OHdG, Nrf2, Keap1, PRDX III and PRDX IV was examined in three separate cohorts: 1) the whole study group; 2) triple-negative tumors; 3) basal-like tumors. Tumors that did not express either steroid receptors or HER2 and were grade II-III and showed ductal histology were classified as triple-negative carcinomas. To further identify the basal subtype among these breast cancer specimens, expression of CK5/6 and EGFR was determined in the triple-negative tumors. Finally, the triple-negative tumors that also expressed both EGFR and CK5/6 were classified as basal-like breast cancers. The main patient and tumor characteristics in each of these groups are shown in Table 2.

**Table 2:** Patient and tumor characteristics.

	Triple-negative	ER+/PR+/HER2-	Basal-like	Total
T				

1	11 (29.7%)	25 (59.5%)	6 (19.4%)	36 (45.6%)

2	23 (62.2%)	14 (33.3%)	22 (71.0%)	37 (46.8%)

3+4	3 (8.1%)	3 (7.2%)	3 (9.7%)	6 (7.6%)

Nodal status				

Negative	21 (56.8%)	23 (54.8%)	18 (58.1%)	44 (55.7%)

Positive	16 (43.2%)	19 (45.2%)	13 (41.9%)	35 (44.3%)

Ki-67				

Negative	3 (8.1%)	7 (16.7%)	2 (6.5%)	10 (12.7%)

Positive	34 (91.9%)	35 (83.3%)	29 (93.5%)	69 (87.3%)

Grade				

I	0 (0.0%)	8 (19.0%)	0 (0.0%)	8 (10.1%)

II	2 (5.4%)	27 (64.3%)	1 (3.2%)	29 (36.7%)

III	35 (94.6%)	7 (16.7%)	30 (96.8%)	42 (53.2%)

The basal-like cohort is part of the triple-negative cohort.

### Statistical analysis

SPSS 17.0.2 for Windows was applied for statistical analysis. The reported p-values are from 2-sided Pearson chi-square tests, except for survival analysis. Survival was analyzed by using Kaplan-Meier curves with the log-rank test and only breast cancer-related death was used as an endpoint. Cox regression analysis was used in multivariate analysis. T-class was divided in statistical analyses to either T1 or larger and grade was divided into either grade I-II or grade III. Probability values below 0.05 were considered significant.

## Results

**Figure 1:**
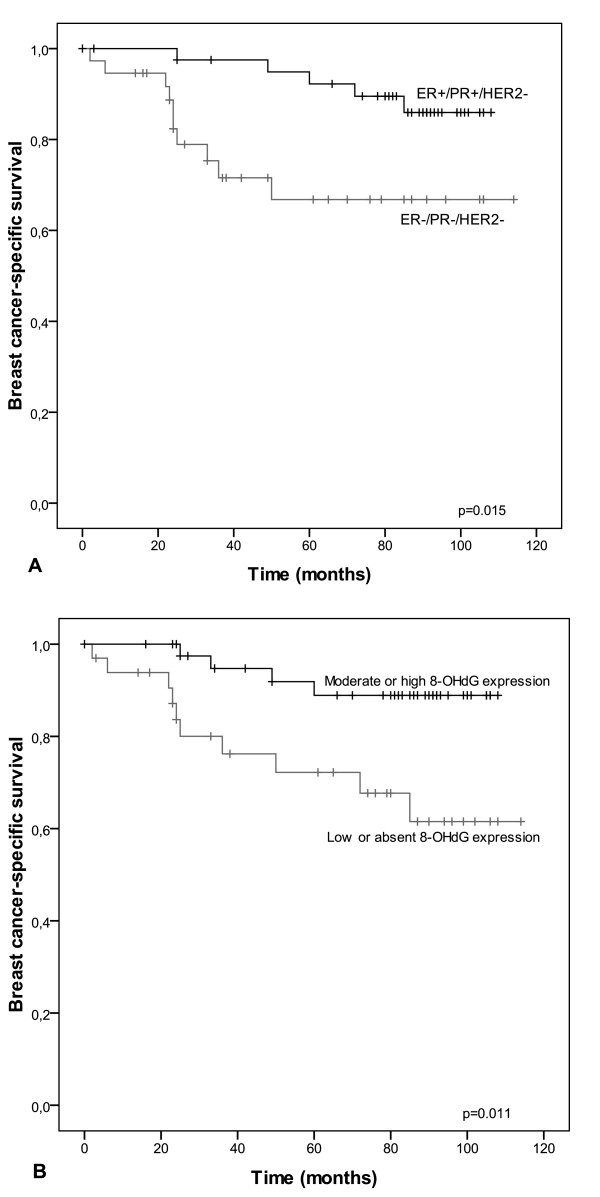
**Kaplan-Meier curves showing breast cancer-specific survival in different subgroups**. TNBC patients have significantly shorter survival compared the control group (p = 0.015) (A). Women with 8-OHdG-positive tumors have better breast cancer-specific survival when all patients are taken into account (p = 0.011) (B).

Thirty-seven of the 79 studied cases were classified as TNBC. Of these 37 TNBCs, 31 (83.8%) exhibited the BLBC phenotype, as they expressed either CK5/6 or EGFR-1. The control cases (n = 42) expressed both ER and PR and were HER2-negative. Primary tumor sizes were larger in TNBC compared to non-TNBC group (p = 0.013) and in BLBC compared to non-BLBC group (p = 0.0054). Patients with TNBC had worse breast cancer-specific survival than the control group (p = 0.015) (Figure 1). There was no difference in survival between the BLBC and non-BLBC groups. The mean follow-up time was 96.6 months.

Cytoplasmic PRDX III expression was observed in 68.4% of cases and nuclear PRDX III in 16.4%. PRDX IV immunostaining was positive in 97.5% of cases and there was virtually no nuclear PRDX IV expression. Nrf2 and 8-OHdG were located mainly in nuclei; Keap1 was mainly cytoplasmic. Immunostaining expression patterns in the subgroups are shown in detail in Table 3.

**Table 3:** Number of positive immunostaining results in different subgroups and corresponding p-values.

	Triple-negative	ER+/PR+/HER2-	p-value	Basal-like	Non-basal-like	p-value
Cytoplasmic PRDX III	3 (8.1%)	9 (21.4%)	0.10	2 (6.5%)	1 (16.7%)	0.42

Nuclear PRDX III	3 (8.1%)	10 (23.8%)	0.060	3 (9.7%)	0 (0.0%)	0.43

Cytoplasmic PRDX IV	27 (73.0%)	20 (47.6%)	0.022	22 (71.0%)	5 (83.3%)	0.53

8-OHdG	13 (37.1%)	30 (73.2%)	0.0016	10 (33.3%)	3 (60.0%)	0.25

Nrf2	12 (33.3%)	11 (26.8%)	0.53	10 (33.3%)	2 (33.3%)	1.0

Keap1	26 (76.5%)	16 (39.0%)	0.0011	22 (78.6%)	4 (66.7%)	0.53

Criteria for positive immunostaining for each antibody are described in the Materials and Methods section.

8-OHdG was overexpressed in the ER+/PR+/HER2- group compared with TNBC samples (p = 0.0016) (Table 3). Keap1 immunostaining was more prevalent in the TNBC group than in the control group (p = 0.0011). Cytoplasmic PRDX III showed a trend towards less frequent expression in the TNBC cohort (p = 0.10). In addition, a lack of nuclear PRDX III expression was more frequently observed in the triple-negative carcinomas compared with the ER+/PR+/HER2- control specimens (p = 0.060). On the other hand, cytoplasmic PRDX IV was overexpressed in the triple-negative breast carcinomas, compared with the non-TNBC group (p = 0.022).

Immunostaining of Nrf2 and Keap1 showed significant co-expression (p = 0.0037). Nrf2 and PRDX III were significantly co-expressed when all cases were considered (p = 0.008) and also in non-TNBC-cases (p = 0.011), but not in the TNBC group alone (p = 0.201). PRDX III expression in nuclei was independent of its presence in cytoplasm. PRDX IV expression was not significantly associated with PRDX III expression (p = 0.068 for expression in the same samples). Expression of 8-OHdG was not associated with antioxidant enzymes when all samples were considered. However, 8-OHdG expression had a significant association with low cytoplasmic PRDX III expression in TNBC cases (p = 0.018), but in non-TNBC cases there was an association with the presence of cytoplasmic PRDX III expression (p = 0.028).

8-OHdG immunostaining was associated highly significantly with the traditional factors of good prognosis (low grade p = 0.00020, low T-class p = 0.00065). Keap1 was overexpressed in grade III tumors (p = 0.0058) and near-significantly in high Ki-67 tumors (p = 0.075). Nuclear PRDX III expression was associated with less aggressive tumor characteristics, since it was overexpressed within the T1 tumor population (p = 0.011) and in grade I-II disease (p = 0.029).

8-OHdG-positive immunostaining was associated with better breast cancer-specific survival (BCSS) when all patients were taken into account (p = 0.01). Keap1-positive patients had shorter BCSS than those with Keap1-negative tumors (p = 0.014). Cytoplasmic PRDX III immunostaining had a nearly significant association with poorer BCSS (p = 0.06). In multivariate analysis none of the studied markers were independent from traditional prognostic factors.

## Discussion

There is great variability in the reported frequencies of triple-negative and basal-like breast cancers in the literature, depending on the criteria used. The prevalence of TNBC has ranged from 17.1% to 30.5% and that of BLBC from 8.0% to 55.7% [13]. Here we reassessed the steroid receptor status and HER2 status of tumors; those tumors without any ER and PR immunostaining were considered as receptor negative and tumors exhibiting more than 10% of invasive tumor cells were considered as steroid receptor-positive. In addition, only grade II-III tumors with ductal histology were included in the TNBC cohort. It is also known that triple-negative and basal-like subtypes of breast carcinoma are not single cohesive entities but instead reflect a collection of different diseases. Outcome in cases of TNBC has consistently been worse compared with ER- and PR-positive tumors, which was again confirmed in the current material. TNBC and BLBC tumors are usually larger and of higher grade compared with receptor-positive breast cancer tumors and some reports suggest that cases of BLBC are more often node-negative [3,14-16]. In the current study, triple-negative carcinomas were larger than ER+/PR+/HER2- cancers, as were basal-like carcinomas compared to non-basal-like triple-negative breast cancers, but no association with nodal status was observed. The proportion of basal-like subtype showing cancers in our material is in line with previous reports [16].

One of the most important estrogen-related carcinogenic mechanisms is oxidative metabolism of estrogen and subsequent formation of ROS [17,18]. Published data from several laboratories suggest that *in vitro*, physiological estrogen concentrations induce significant oxidative stress and that estrogen-induced ROS formation takes place in mitochondria in particular [19,20]. Other studies have provided evidence that 8-OHdG levels in the ER-positive MCF-7 breast cancer cell line are over 9-fold higher than in triple-negative MDA-MB-231 cells [9] and inhibition of estrogen receptor alpha expression significantly reduces estrogen-induced 8-OHdG formation in MCF-7 cells [21]. Since estrogen levels in ER-positive tumors are higher than in ER-negative ones [22], our finding that 8-OHdG (as a marker of ROS-derived DNA damage) was highly overexpressed in the ER+/PR+/HER2- group (73.2%) compared with TNBCs (37.1%) is in line with the above data. Previous and current data taken together suggest that ROS play a major role in steroid receptor-positive breast cancer pathogenesis in particular, but not necessarily in cases of TNBC (including BLBC).

A negative prognostic value of immunohistochemical 8-OHdG expression has been reported in connection with at least colorectal carcinoma [23], ovarian cancer [24] and malignant melanoma [25]. Nevertheless, there is a growing body of evidence that in breast cancer the prognostic value of 8-OHdG is different. We have recently reported that immunohistochemical 8-OHdG expression is an independent factor related to good prognosis in breast cancer, especially as regards ductal histology [26]. In another previous study we found that 8-OHdG expression was significantly diminished in invasive breast carcinomas compared with non-invasive breast lesions [27]. The current data confirm previous results, as 8-OHdG was a marker of good BCSS also in the current population.

When cells are exposed to oxidative stress, Keap1 undergoes a modification that allows Nrf2 to be released from a complex with it and translocate to the nucleus where it binds to antioxidant response elements of DNA [8]. Nrf2-mediated antioxidant enzyme induction is one of the major defense mechanisms against excessive ROS production and, on the other hand, PRDX enzymes are considered to be among the most efficient cell redox state-regulating enzymes [8,28]. The importance of PRDXs is derived partly from their wide subcellular distribution, in contrast to most other antioxidant enzymes. The majority of ROS are produced in mitochondria under physiological conditions and PRDX III is an especially important part of antioxidant defense, since it is located mainly in mitochondria. Peroxiredoxin IV is found in lysosomes, peroxisomes and the endoplasmic reticulum, where oxidative stress is also a potent threat [5]. Previous studies carried out *in vitro *have demonstrated induction of PRDXs via the Nrf2/Keap1 pathway [28], but there are no reports on Nrf2 or Keap1 in clinical breast cancer material. The current data is in line with previous *in vitro *results as regards steroid receptor-positive breast cancer, since there was highly significant co-expression of PRDX III and Nrf2 in ER+/PR+/HER2- cases, which may reflect Nrf2-mediated PRDX induction after estrogen-induced oxidative stress. Furthermore, PRDX III was associated with 8-OHdG expression in the non-TNBC cohort, probably representing antioxidant induction as a response to oxidative imbalance. Keap1 was highly overexpressed in the TNBC group compared with the steroid receptor-positive control group, which implies that there is no need for intensive (Nrf2-mediated) free radical scavenging in cases of TNBC as a result of a lack of estrogen-induced oxidative stress. Keap1-positive tumors were more aggressive than Keap1-negative ones and Keap1 expression associated also to poor prognosis. This probably derives from the sensitive induction of Keap1 in stressed and damaged tumors, rather than carcinogenesis promoting features of Keap1 itself. However, further mechanistic investigations are required to confirm these hypotheses.

In a previous tissue microarray study we reported on PRDX III overexpression in ER- and PR-positive breast cancers and PRDX IV in PR-positive cases [10]. In that study, with unselected breast cancer cases and with older methods of steroid receptor assessment, PRDX III- and PRDX IV-positive cases were associated with better prognosis. In the current material neither of the studied PRDX enzymes showed significant association with survival. We observed PRDX IV overexpression in TNBC, especially in non-basal-like breast cancers. PRDX III expression was similar in the TNBC and non-TNBC groups, but PRDX III-positive tumors tended to be smaller and of lower grade. This association with lower grade was observed in a previous study [10] and suggests a protective role of this mitochondrial enzyme in breast carcinogenesis. All in all, PRDXs III and IV could function as protective enzymes in ER- and PR-positive breast carcinomas, working against the ROS induction of estrogen metabolites.

## Conclusions

To summarize, the current results, together with previous preclinical observations, suggest that estrogen induces significant oxidative stress in ER+/PR+ breast cancer compared with TNBC. The Nrf2/Keap1 pathway is more active in steroid receptor-positive disease and this subsequently causes induction of antioxidant defense that is not observed in TNBC. Further studies with larger patient groups are required to elucidate possible prognostic roles of the studied factors.

## Competing interests

The authors declare that they have no competing interests.

## Authors' contributions

PK participated in the design of the study, contributed to the evaluation of immunostainings, performed the statistical analysis and wrote the different stages of the manuscript. SK helped in study design, evaluated immunostaining results, coordinated steroid receptor and HER2 assessment and provided comments on drafts of the manuscript. YS participated in the design of the study and immunostaining evaluation, participated in data interpretation and critically analyzed the manuscript. AJV contributed to study design, the collection of patient material and was also responsible for manuscript preparation. All authors read and approved the final manuscript.

## Pre-publication history

The pre-publication history for this paper can be accessed here:

http://www.biomedcentral.com/1471-2407/11/262/prepub
